# Is no news good news? Inconclusive genetic test results in *BRCA1 *and *BRCA2 *from patients and professionals' perspectives

**DOI:** 10.1186/1897-4287-8-1

**Published:** 2010-01-12

**Authors:** Audrey Ardern-Jones, Regina Kenen, Elly Lynch, Rebecca Doherty, Rosalind Eeles

**Affiliations:** 1 The Royal Marsden NHS Foundation Trust, Downs Road, Surrey, UK; 2 The College of New Jersey, Department of Sociology and Anthropology, New Jersey, USA; 3 Austin Hospital, Melbourne, Australia; 4 The Institute of Cancer Research, 15 Cotswold Rd, Surrey, UK

## Abstract

**Background:**

Women from families with a high risk of breast or ovarian cancer in which genetic testing for mutations in the *BRCA1/2 *genes is inconclusive are a vulnerable and understudied group. Furthermore, there are no studies of the professional specialists who treat them - geneticists, genetic counsellors/nurses, oncologists, gynaecologists and breast surgeons.

**Methods:**

We conducted a small qualitative study that investigated women who had developed breast cancer under the age of 45 and who had an inconclusive *BRCA1/2 *genetic diagnostic test (where no mutations or unclassified variants were identified). We arranged three focus groups for affected women and their close female relatives - 13 women took part. We also interviewed 12 health professionals who were involved in the care of these women.

**Results:**

The majority of the women had a good grasp of the meaning of their own or a family member's inconclusive result, but a few indicated some misunderstanding. Most of the women in this study underwent the test for the benefit of others in the family and none mentioned that they were having the test purely for themselves. A difficult issue for sisters of affected women was whether or not to undertake prophylactic breast surgery. The professionals were sensitive to the difficulties in explaining an inconclusive result. Some felt frustrated that technology had not as yet provided them with a better tool for prediction of risk.

**Conclusions:**

Some of the women were left with the dilemma of what decision to make regarding medical management of their cancer risk. For the most part, the professionals believed that the women should be supported in whatever management decisions they considered best, provided these decisions were based on a complete and accurate understanding of the genetic test that had taken place in the family.

## Background

In an investigation of psychosocial aspects of genetic counselling and testing, Vadaparampil et al (2004) concluded that a key area deserving research and clinical attention was the area of inconclusive test results [1]. Members of hereditary breast/ovarian cancer (HBOC) families who have been affected by cancer are offered testing for mutations in the *BRCA1 *and *BRCA2 *cancer predisposition genes with the hope of identifying the cause of the family's cancers. This can then provide information for others in the family about their own individual risk. In the majority of cases, no pathogenic mutation is identified [2]. This may mean that the tested family member developed cancer by chance, or there is a very small chance that a mutation is present in *BRCA1 *or *BRCA2 *but was missed due to limitations in current technology. Alternatively, the individual may have a mutation in a gene, or genes yet to be identified. Currently, genetic testing does provide information on variants of unknown significance (VUS). However, in our small study, no variants had been detected on our group of tested breast cancer patients.

The fact that there is no identified pathogenic mutation in a *BRCA *genetic test may pose problems for the woman and the provider. Both may need to make decisions based on uncertain information. For example, it is already known that a breast cancer attributable to a mutation in the *BRCA1 *gene may have different tumour characteristics, different recurrence risks and possibly different treatment implications [3-6]. One of the reasons that women disclose their positive *BRCA *genetic test result is to provide genetic risk information to their sisters or daughters. But in the case of an inconclusive result, they can only rely on their family history, and have no genetic test to offer their kin. How does this make the woman feel? There is limited information that focuses specifically on women receiving an inconclusive genetic test result [2,7,8].

Patenaude et al [9] and Gadzicki and Wingen [10] found that women with inconclusive results reported their results less frequently to their relatives than did women with a positive result [1,10]. Furthermore, even though a large majority of tested women told their sisters their genetic results, fewer did so when the results were inconclusive [9,11]. Research findings have differed with respect to the implications of receiving an inconclusive result. Those who received an inconclusive, rather than a positive result, said that they were more worried about cancer, less relieved by their test result and felt that their quality of life had been more adversely affected by it [12]. Another study concluded that women with a high familial risk but with no identified mutation appeared to be reassured after disclosure and showed the same levels of worry and distress after genetic testing as did women who were identified as *BRCA *carriers [2]. Yet, O'Neill et al [8] found that women who had an intolerance of uncertainty were at risk for long-term distress. Maheu and Thorne [13] reported the effect of uninformative *BRCA *results in another small qualitative study. Women made sense of their test results by drawing on their own understanding of the patterns of breast and ovarian cancer that was in their families and subjectively assumed that they were either carriers or not. Another study looking at the long term effect of *BRCA *genetic testing on women noted that the majority of 147 women with inconclusive genetic test results were relieved not to be identified as a mutation carrier, although this relief was not as great as that felt by women receiving a true negative predictive test result [14]. More recently, Cypowyj et al [15] found that the majority of women with inconclusive results felt unsure as to what this result really meant.

We could not find any peer-reviewed articles on the investigation of the roles of health-care professionals who interact with women facing the uncertainty inherent in an inconclusive genetic test result. One study that does look at the role of the professional with regard to advising women with known *BRCA *mutations about preventive surgery [16] suggests that *BRCA *carriers also have dilemmas related to decision making about different prevention options, though, more is known about their future risk. Another related article by Matloff et al [17] examined what cancer genetics specialists predicted that they would do themselves if they were at 50% risk of carrying a *BRCA1 *or *BRCA2 *mutation. To the best of our knowledge, ours is the first study to investigate the experience of both the clients from a cancer genetic risk clinic and the professional providers that treat them within the same institutional social context.

## Methodology

We conducted a qualitative exploratory study at a major cancer centre in the UK. A qualitative research design was chosen because of its ability to capture complexity and process and the meaning attached to individual action [18]. The researchers used an inductive process involving coding and identification of recurrent patterns, relationships and processes in the data, which emphasises the participants' own accounts of their phenomenological and social world [19-21]. This approach does not assume that there is only one measure of reality that can be objectively grounded [22,23]. We used both individual interviews and focus groups to gather as much rich data as possible. The most important feature of focus groups is the ability to gather in-depth responses as part of the interaction between the group participants [24,25].

We chose focus groups as the methodology for the women because we knew from clinical experience that the topic was an emotional one and that the interactions between the women ought to reveal richer information than could be gathered through individual interviews. Any differences between the interviews of the professionals and the focus groups should be minimal because focus groups are group interviews [26]. The focus group and individual interview guides were similar in that both asked general questions, followed by probes on topics of interest described below and thus the findings can be compared.

### Focus Groups

Letters of invitation were sent to 16 women who had undergone *BRCA1 *and *BRCA2 *mutation analysis through a cancer genetics clinic at a major cancer centre in the UK. All these women had received written notification informing them of their "inconclusive" genetic test result. All had experienced a diagnosis of breast cancer under the age of 45, and had a living daughter and/or a sister without cancer over the age of 18. We offered the women a choice of three dates. Two women brought one sister to the focus group, one woman brought two sisters and one woman brought a daughter. A total of 13 women participated. All the groups included women from different families. Four women contacted us to say that they were unable to attend on the dates proposed. The other four did not respond even though we attempted to re-contact them by telephone. If an individual was known to be currently unwell and receiving treatment, they were not approached. All the women signed informed consent forms.

Because of the importance of this subgroup of women from HBOC families and their health-care professionals who care for them, we investigated reactions to inconclusive *BRCA1/2 *test results in both women from high-risk families and professionals who practice in a large cancer centre. We examined several issues: 1) how women from these types of high-risk families who have developed breast cancer under the age of 45 cope with the uncertainty of developing a second primary breast or ovarian cancer in the future; 2) how their female relatives interpret and use these inconclusive results; 3) whether this group are treated differently by health professionals (as compared with those without a family history or those definitively shown to carry a *BRCA1 *or *BRCA2 *mutation) in terms of surveillance advice and recommendations for prophylactic surgery; and 4) health professionals' feelings about delivering inconclusive genetic test results and issues in counselling these women and whether this uncertainty affects the patient doctor relationship.

We used a semi-structured moderator's guide with open-ended questions. Questions and probes were asked relating to: dealing with uncertainty; regrets (if any) about being tested for a genetic mutation; how relationships and expectations have changed since their cancer diagnosis; the effect of the passage of time; belief in science and technology; attitudes towards health care professionals; and family feelings about inconclusive results.

### Interviews with health care professionals

The 14 professionals at the cancer centre who worked with women affected by, or at high familial risk of, breast and ovarian cancer were invited by letter to participate in the study. Only two refused, stating time constraints. We therefore interviewed 12 professionals including staff from the following specialties: clinical genetics, oncology, cancer genetic nurse counselling, genetic counselling, gynaecological surgery and breast surgery. The interviews, lasting from 15 to 30 minutes, were conducted in the cancer centre.

Our purpose was to ascertain the professionals' perspectives on health behaviours and prophylactic measures and their experiences with women in this situation, including their perceptions of the women's attitudes and feelings as well as their own feelings. All the professionals provided written informed consent.

We used an open-ended, semi-structured interview schedule and asked specific questions about: the professionals' experiences with women who had an inconclusive *BRCA1 *and *BRCA2 *genetic test result; how they dealt with the uncertainty raised by an inconclusive result; their medical management advice for these women and the reasoning behind the advice; whether they believed that the women understood what an inconclusive result was and how they endeavoured to ensure accurate comprehension; whether they thought there was disagreement among different specialists about the medical management of these women; and the professionals' own emotional reaction to providing an inconclusive result.

RK, EL, and AAJ analysed transcripts of the focus group sessions and interviews for recurring themes after repeated close reading of the material. They separately read and reread the focus group and interview transcripts, noted each theme presented by the respondents and then compared and discussed their interpretations. There was close agreement on the main themes. Direct quotes are used throughout the paper to validate the findings. The focus groups and interviews were tape-recorded with consent from all the participants and the recordings were transcribed. Only pseudonyms were used. The Hospital's Clinical Research Committee and Local Regional Ethics Committee approved the study. The authors confirm that there is no conflict of interest between themselves and others that might bias their work.

## Findings: Participants

Several themes emerged from the focus groups. We discuss the main ones in more detail below.

### Genetic Testing for the Benefit of Family Members

Most of the women in this study underwent the test for the benefit of others in the family and none mentioned that they were having the test purely for themselves. In fact, four of the eight affected women said that they would not have had the test at all if they did not feel that it could provide helpful information for other relatives, particularly sisters, daughters or nieces.

"I did this for the future generations. I wasn't worried about...the knock-on effect of me, I was already there."

(Caroline, affected by cancer)

### Implications of the Inconclusive Results

While the women seemed to accept the inconclusive result as leaving them, as one woman put it, "back to square one", many of them were not sure how to process the inconclusive result or had a misunderstanding at some level. Julie said that it was "sort of good news", but added, "isn't it, I think". Caroline deeply believed that her cancer was caused by a genetic mutation, but hoped that a positive genetic test might lead to prevention of any further mutation, a scientifically flawed assessment. As indicated in the following quote, Monica also misapprehended her situation.

"... *of all the genes that one could pick up I'd far rather it was breast cancer than so many other things which are really, really so much more horrendous and difficult to deal with. Because I think, you know, it's quite a gentle gene..."*

(Monica, affected by cancer)

### Management of Cancer Risk

The majority of the women felt that an inconclusive result allowed them to avoid making a choice about prophylactic surgery, something they might have considered more strongly if a mutation had been identified.

"I know it's harder for my sister to make a decision....as I said, I'd sort of made my mind up anyway, that it was inherited, so whether it came back as positive, then it would have made a difference for my sister who would've, I think, probably gone down the line of having a prophylactic mastectomy if it had definitely been positive now."

(Mary, affected by cancer, referring to sister not present)

### Communicating inconclusive genetic test results

#### Telling adults

It is usual practice for a letter to be given to an individual who carries a gene mutation to distribute to other relatives informing them about the meaning of the test result, the screening recommendations and how to arrange referral to the clinic. It is not as common to provide written information for family members of those receiving inconclusive results. The women usually did not tell relatives outside the immediate family about their inconclusive *BRCA1/2 *mutation test result. They felt that an inconclusive test result did not provide any further information and might worry them unnecessarily. A few of the women had particular difficulty in telling their mothers. Kim reported that her mother always changed the subject so she did not press her, while Monica tried to protect her mother.

"I think past a certain point it's very complicated for...for older people ...there's a feeling of guilt there. ...I think there's no point in clouding their lives, really. I mean my mother's just had a stroke, I wouldn't dream of mentioning to her that we're here discussing a gene that may have been passed on. I cannot see the point, I think it's a perfectly negative, unkind thing to do."

(Monica, affected with cancer)

#### Telling Children

Some participants felt that there was no need to tell their children about inconclusive results. They said, however, that if a *BRCA1 *or *BRCA2 *mutation had been detected, they would have told their children. It appears from the quotes above and below that many of the women felt that an inconclusive result gave them a reprieve from having to be the bearer of bad news to the family.

"I think they [the older children] presumed that if it was positive I would say something. And because I haven't said anything, it's either negative or it's inconclusive. And I did say there was a high chance it would be inconclusive."

(Pat, affected by cancer)

## Findings: Professionals

### Understanding and explaining inconclusive genetic test results

Geneticists, oncologists and genetic nurses/counsellors are the health professionals that most often discuss risk with women from families with a hereditary cancer diagnosis. They frequently find that the women they meet with do not have a clear grasp of what an inconclusive genetic test result means despite undergoing genetic counselling prior to having testing, as well as receiving a letter following an appointment.

*"I think it's one of the most difficult situations that you have. So I think sometimes an uninformative result can be associated with...anxiety on the part of the patient and also on the part of the doctor, because we like to deal with absolutes and I'm not sure that the medical profession's always very good at dealing with uncertainty*."

(Dr D, oncologist)

The surgeons were more concerned that the women were educated about their general cancer risk and risks related to surgery.

.... *there's much that is unknown about this, and therefore we come back to making individualised decision-making on the basis of the knowledge of the present tumour, the risk-benefits of risk-reduction surgery against the background of dying of breast cancer*.

(Dr J, Breast surgeon)

### Views on future management after inconclusive genetic test results

Bilateral prophylactic mastectomy has been found to reduce the risk of a future breast cancer for a woman carrying a *BRCA1 *or *BRCA2 *mutation by as much as 90% [27]. The question of prophylactic surgery, however, becomes much more problematic in high-risk women who have an inconclusive result. The professionals worry about the timing of surgery or that the women might learn in future, because of progress with testing technology that they do not, in fact, carry a mutation and therefore had unnecessary surgery. But some women who had seen most female members of their families develop breast cancer wanted the procedure, regardless of the test results, for peace of mind. Dr H, a surgeon, was particularly worried about these women.

"*I don't think prophylactic surgery is for everybody...although if you've definitely got the gene then fair enough, you know, you can give them statistics, but to do it on an inconclusive test, I really think it's putting them at risk of major psychological problems."*

(Dr H, breast surgeon)

The professionals also felt uncomfortable about a woman choosing prophylactic surgery in the case of an inconclusive result unless the family history was extremely severe.

"There are some women who are absolutely adamant that they want radical surgery immediately when they have an inconclusive results and I would very much err on the side of caution. There are, however, some women who, with a really horrible family history where their risk is obviously very great, who will not consider surgery in any circumstance, where my own view might be that I would encourage them to do so. Obviously in genetic counselling we are totally nondirective but I think inevitably your own sort of feelings and your own point of view come through to a certain extent, I think it's foolish to try and sort of, um, say that we don't..."

(Dr A., geneticist)

### Agreement between professional specialties

Opinions varied regarding whether there were differences in management advice between professional specialties. A few of the professionals believed that the different training among specialists provided slightly different lenses to interpret the same finding. Dr B, an oncologist, said that from his and other oncologists' experiences, there were differences between geneticists, oncologists and surgeons.

".... and it's interesting, actually, because I do think some oncologists are more pro-prophylactic surgery than some geneticists, that's certainly been my experience. And that may be because ...it's been coloured by their experience of looking after young people with cancer."

(Dr B, oncologist)

### Emotional reactions to providing inconclusive genetic test results

Nine of the twelve professionals said that they found discussing inconclusive genetic test results with women at high risk for breast and ovarian cancer to be difficult. They used many different words to discuss these feelings. They reported that they felt "uneasy", "frustrated", "anxious", "stressed", "inadequate" or "disappointed" because science still had not developed at test that could give the women a definitive result. One oncologist reported that sometimes when he could not provide an answer, it made him feel that he was a failure in some way. These types of feelings were most strongly expressed by the professionals working in the genetics clinic whose job it was to provide and explain genetic test results.

"...I think it makes you feel a little bit anxious, that it shifts the power, sort of, in the relationship. You're not the person who has all the answers, you're having to start sentences with words like "well, we don't know...we don't know exactly what this means for your family" or "we can be less certain about the risks in your family"...and you know sometimes I think it can make you feel that you're a failure in some way and that you can't provide an answer."

(Dr D, oncologist)

## Discussion

Women from HBOC families who receive inconclusive *BRCA *test results are in a unique situation; their management and psychological issues are different from both women in the general population and women who are known to carry a gene mutation. They may also be identified with a VUS (variant of unknown significance) that may in the future change with time. They do, however, share with the women in families in which a *BRCA1 *or *BRCA2 *mutation has been identified a fear of a new cancer and the overwhelming experience that comes along with that cancer [28]. The women in this study who had had testing and a diagnosis of breast cancer wanted genetic testing mainly for the benefit of others in their family, but receiving an inconclusive test result left them with little information to impart.

Most of the affected women in this study felt relieved that an inconclusive genetic test relieved them of having to choose between unpleasant options about their future medical management. They also felt relieved of the responsibility of sharing information with more distant members of their families because they did not believe that telling them about an inconclusive result would be meaningful and might be worrisome. These results conflict with those of Cypowyj et al [15]. Some of the participants in their study felt sure they were carriers. Patenaude et al [9] also found that women who received inconclusive results less frequently conveyed these results to even their first-degree relatives. However, in a small, cross-sectional descriptive study, Loescher et al [29] found that women who had uninformative test results were concerned about screening needs for family members.

The affected women and their unaffected sisters/daughter who participated in the focus groups were a self-selected sample. Relationships between the affected woman and the family member/s who attended were evidently good enough for them to attend together. Several authors have indicated the support that sisters can provide [11]. In our focus groups, there was wide variation in sisters' interpretations of an inconclusive genetic test and feelings about what preventive measures might be taken in the future, some placed more emphasis on the lack of clarity in the results than others. The presentation of test results can be confusing. Frost et al [30] recommended that both patients and health professionals needed to ensure that they used a common vocabulary because "uncertain" can refer to both results or outcome. The variability we found reflects the variability found in the literature [2,7,12,31]. One of the reasons for this variability might be due to individual differences in risk perception [32].

The professionals were very open about their opinions, perceptions and feelings. They felt that members of different specialties might view the women's risk situation differently due to difference in their training, the kinds of patients they most commonly saw. The findings that were the most interesting were those related to the professionals' emotional reaction to delivering inconclusive genetic test results. The professionals appeared to worry more about the interpretation of inconclusive results than the women did about receiving them, and many of the specialists found it difficult to feel assured that their patients left the counselling session with an accurate understanding of the meaning of an inconclusive result. Feelings of stress, disappointment and anxiety were expressed primarily by those working in the genetic clinic whose task it was to present the inconclusive results to the women. The breast surgeons, however, were less affected as they tended to work from the premise that the women had received sufficient and accurate information from the genetics clinic.

The professionals also were not sure about how to explain accurately what an inclusive genetic test result meant to extended kin. Hallowell et al [33] found that the women in her study required clear information about the meaning and implications of the different types of test results because some of them interpreted an inconclusive result as definitive confirmation that a cancer-predisposition mutation was not present within the family. Dorval et al [12] concluded that it was important for the health professionals to fully impart to women that an inconclusive result does not preclude the possibility that women in the families with an inconclusive genetic test may still be at a higher risk of developing breast and ovarian cancer.

### Limitations

There are several limitations to this exploratory study. Firstly, the sample is small. Also, all the participants described their relationship with the relative who attended as close; those who were not close may well have responded differently. Furthermore, the study location may be atypical because it is a major research cancer centre rather than a district hospital. Women who developed breast cancer and were treated in a district hospital may not have had similar experiences because of the differences in the location of genetic services. It is a retrospective study and selective memory may act as a bias. Also, participation in a focus group and hearing opinions of others may change individual women's perspectives and not reflect their opinions prior to their participation. Members of focus groups react to comments of other participants. Individual interviews are conducted privately and while the interviewees cannot be impacted by the opinions of others, they also may forget to mention important attitudes that might have been elicited through others' statements.

Despite these limitations, we feel that the findings of this study are important because it is the first study that we are aware of that looks at both the health professionals' and women's cognitive and emotional perspectives related to the receipt of an inconclusive *BRCA1 *and *BRCA2 *mutation detection test. This research also provided ideas for improving follow-up care for these women from HBOC families.

### Suggestions for improved care

Based on the themes that emerged from this study, it would seem that the needs of women who have received inconclusive results are mostly, but not always, met. Usual practice in this particular clinic is for women with inconclusive results to receive their results by letter, with an offer of a consultation should they wish to discuss the result further. Hallowell et al [33] recommended that all persons who were informed that they had an inconclusive genetic test result should have a follow-up session with a genetic clinician either by telephone or in person, although this may be limited by resources within a publicly-funded institution.

It maybe helpful to develop services such as study days, specifically for women who have received uninformative *BRCA *test results such as study days. A newsletter may be another option to update people who have undergone genetic testing. Increasingly, health websites have been a resource and support for women at high risk. A major one is FORCE (Facing Our Risk of Cancer Empowered) [34]. We would also suggest that easy-to-read leaflets be designed that could be handed out to extended kin with up-to-date scientific explanations on genetic testing as well as management options and contact numbers that could be accessed by both the families and other health professionals. Annual telephone contact could provide them with an update on new issues relating to their family history and genetic testing status e.g. new screening modalities for high risk women where there is no identified mutation. Some of these women may be being screened in an outreach clinic where professionals are not immediately aware of new developments in the genetics field.

Even though genetics professionals had a clear understanding of the scientific basis of an inconclusive *BRCA *genetic test result, they expressed frustration when imparting this information in light of the uncertainty involved. Perhaps workshops addressing how to cope with this frustration should be offered at professional meetings. It is also imperative to provide up-to-date genetic information to other health-care professionals. These can be arranged as part of 'update' study days as well as through written or web-based communication.

Clinicians at large institutions often benefit from practicing as part of an interdisciplinary team. Health professionals caring for women who have had an inconclusive genetic test need to be updated continuously with regard to new scientific discoveries; for example, in the interpretation of tumour histology and its impact on the probability of an association with a germline mutation [4]. It is essential that formal or informal links between geneticists and other professionals caring for these patients be formed.

## Conclusions

This study has highlighted the complex feelings of women who find it hard to make decisions with regard to the management of their own health in the face of uncertainty, their fears for their own futures, as well as their concerns for their families. Some of the members of the medical teams who manage these HBOC families who have received inconclusive *BRCA *test results also experienced emotional difficulties and feelings of inadequacy and unease when discussing prevention options due to state of current knowledge in the field. Instituting the suggestions made above may go a long way in ensuring that women from HBOC families with an inconclusive *BRCA1 *and *BRCA2 *mutation result receive information about the latest scientific advances and medical management options.

## Competing interests

The authors declare that they have no competing interests.

## Authors' contributions

AAJ, RK and RE conceived and designed the study. AAJ and RK and EL analysed the data. RK and EL interviewed the professionals. AAJ and RK and EL moderated, recorded and took notes of the focus groups. RD transcribed the interviews and contributed to the organisation of the study. All authors read and approved the final manuscript.
